# T150. REAL-TIME FMRI NEUROFEEDBACK TO DOWN-REGULATE SUPERIOR TEMPORAL GYRUS ACTIVITY IN PATIENTS WITH SCHIZOPHRENIA AND AUDITORY HALLUCINATIONS: A PROOF-OF-CONCEPT STUDY

**DOI:** 10.1093/schbul/sby016.426

**Published:** 2018-04-01

**Authors:** Natasza Orlov, Vincent Giampietro, Owen O’Daly, Gareth Barker, Katya Rubia, Philip McGuire, Sukhi Shergill, Paul Allen

**Affiliations:** 1 Institute of Psychiatry, Psychology & Neuroscience, King’s College London; 2 Cognition Schizophrenia and Imaging (CSI) Lab; 3 University of Roehampton

## Abstract

**Background:**

Neurocognitive models and previous neuroimaging work posit that auditory verbal hallucinations (AVH) arise due to increased auditory cortex (AC) activity and altered connectivity between the AC and other speech and language regions [e.g. 1]. In the present study we examined if patients with schizophrenia (SCZ) and AVH could be trained to down-regulate AC activity using real time functional Magnetic Resonance Imaging neurofeedback (rtfMRI-NF) [2]. We also examined the effects of rtfMRI-NF training on functional connectivity between the AC and other speech and language regions.

**Methods:**

Eleven patients with SCZ (Table 1) and treatment refractory AVH were recruited to participate in the study and were trained to down-regulate auditory cortex (AC) activity over an average of fourteen rtfMRI-NF runs conducted during a two-week training period (Fig 1). We used a functional localiser to identify the speech sensitive superior temporal cortex (STG) (Figure 2A). At the end of the training period, AC activity, functional connectivity and AVH symptom levels were compared pre and post training.

**Results:**

Patients successfully learnt to down-regulate activity in their AC over the rtfMRI-NF training period. Post training, patients showed increased connectivity between the AC, the inferior prefrontal gyrus and the inferior parietal lobe Figure. There was also a modest reduction in AVH symptom levels post compared to pre training (Table 2).

**Discussion:**

The AC is as suitable target region for rtfMRI-NF in patients with SCZ and treatment refractory AVH. Successful down-regulation of AC activity can increase functional connectivity between speech motor and perception regions. These findings raise the possibility that rtfMRI-NF training could be used as a novel therapeutic intervention in this clinical population.

